# A Case of Gun-shot Wound of the Head

**Published:** 1873-10-15

**Authors:** E. Powell, D. A. K. Steele

**Affiliations:** House Surgeon


### Hospital Reports.

### SURGICAL WARDS COOK COUNTY HOSPITAL.

A CASE OF GUN-SHOT WOUND OF THE HEAD. SERVICE OF PROF. E. POWELL. REPORTED BY D. A. K. STEELE, M.D., HOUSE SURGEON.

Sept. 13th, 1873.—C. B——, aged 17; bar-tender; native of Germany; was admitted to hospital, with gun-shot wound of head, received in a saloon affray about two hours previous to admission. On examination, find a gun-shot wound of forehead, one and a half inches above the left eyebrow, penetrating frontal bone and lodging in brain substance. The ball appears to have entered in an oblique direction, passing across into right hemisphere of brain. Patient very restless, and apparently suffering a good deal; is conscious most of the time; pupils dilated; pulse 60, and weak; respiration slow and labored. Gave morphia sulph., gr. one-third, hypodermically, and applied cold compress to wound.

Sept. 14th, 9 A.M.—Pulse 56. Patient restless all night; semi-conscious this morning. 8 P.M.—Pulse 58; is more rational; vomited a considerable amount of dark, bilious matter during the day. Ordered bland, nutritious diet at regular intervals—milk, beef-tea, etc.

Sept. 15th.—Bowels not having moved, ordered the following:

℞.—Hydrarg. chlor. mit., gr. x.
Soda bicarb., ℈j.
Misce. Fiat chart. ij.
Signa. To be taken two hours apart.

Sept. 16th.—Pulse 60; had a free movements of the bowels; feels better.

Sept. 17th. — Pulse 58; two stools to-day; complains of general pain in the head; also, fullness; says: "head feels like bursting." Ordered the following mixture, to relieve pain:

℞.—Potass. brom., ʒ vi.
Quiniæ sulph., gr. xxiv.
Syrupi.
Aquæ, aa. f. ℥ ij.
Misce.

Signa. Tablespoonful every four hours.

Sept. 18th. — Pulse 62; slight purulent discharge from wound. Removed several small pieces of bone that were lying loose in the comminuted opening of skull.

Sept. 19th. — Pulse 60; respiration 18; temperature 98; much improved; mind quite clear; conversed rationally on a variety of topics; appetite better; took a little solid food, for the first time since the injury.

Sept. 20th, 9 A.M.—Pulse 64; temperature 97. Removed depressed piece of bone lying on dura mater, about one-half inch in diameter, and three small pieces of the bullet, weighing four grains, that had been torn off the bullet in its passage through the skull. 8 P.M. — Pulse 58; urine contains a large quantity of phosphatic deposits.

Sept. 21st, A.M. — Pulse 64; patient sat up a short time. P.M. — Pulse 56; pupils normal.

Sept. 22d, A.M. — Pulse 64; quite cheerful; says he will soon go home. P.M. — Pulse 60.

Sept. 23d, A.M.—Pulse 62; extremities cold. P.M. — Pulse 60; complains of intense pain in right occipital region. Gave morphia sulph., gr. one-third, in addition to the bromide of potash mixture.

Sept. 24th, A.M.—Pulse 64; respiration 16; temperature 96; was delirious all night; pupils dilated; extremities cold; tongue thickly coated white; no appetite; great thirst. P.M. — Pulse 60; bowels moved to-day.

Sept. 25th—Pulse 64; rational; pain in occiput.

Sept. 26th, A.M. — Pulse 80; respiration 20; temperature 97; unable to swallow; paralysis of pharyngeal muscles and bladder; breathing slow and stertorous; trachæ obstructed by purulent sputa; died at 6 P.M.

*Autopsy eighteen hours after death.*—Scalp over vertex ecchymosed. Gun-shot wound of head: ball entered one and a half inches above left eyebrow; orifice through frontal bone about half an inch in diameter (not stellated); at lower and external border of orifice there was an oblong, slightly depressed piece of bone, about one-fourth by one-half inch in size. Membranes of brain very much congested; the vessels all engorged with dark, venous blood, standing up promptly. No effusions outside of dura mater; considerable effusion beneath it. Ball entered left hemisphere, about half an inch to left of longitudinal fissure, passing, in an oblique direction, backwards across fissure, entering right hemisphere, and continuing its course downwards and backwards, striking skull-wall about an inch above right squamo-parietal suture, and deflecting back into right hemisphere, without lacerating the membranes, or fracturing the table of skull, passed on lodging about center of right hemisphere, one inch from surface of brain. The track traversed by the ball was about five and a half inches in length, and lined by a pyogenic membrane. The brain substance, within a radius of an inch and a half of the track of the ball was very much congested and inflamed. In several places along the track, large clots of blood were found. Left hemisphere was comparatively healthy, and normal in appearance. Considerable effusion in all the ventricles; a few small patches of lymph were found near entrance of ball, and at the point of deflection. Brain was rather soft; all the other organs were in a healthy condition.

